# Right axillary and femoral artery perfusion with mild hypothermia for aortic arch replacement

**DOI:** 10.1186/1749-8090-9-94

**Published:** 2014-05-28

**Authors:** Jige Guo, Yue Wang, Jihong Zhu, Jie Cao, Zili Chen, Zhijun Li, Ximing Qian

**Affiliations:** 1 Department of Cardiothoracic Surgery, Sir Run Run Shaw Hospital, College of Medicine, Zhejiang University, No. 3 Qingchundong Road, Hangzhou 310016, China; 2 Department of Anesthesiology, Sir Run Run Shaw Hospital, College of Medicine, Zhejiang University, Hangzhou 310016, China

**Keywords:** Aortic arch surgery, Cardiopulmonary bypass, Mild hypothermia, Brain protection, Selective antegrade cerebral perfusion

## Abstract

**Objectives:**

Aortic arch replacement is associated with increased mortality and morbidity especially in acute type-A aortic dissection. Although hypothermic circulatory arrest with selective antegrade cerebral perfusion has been widely used because of its excellent cerebral protection, its optimal perfusion characteristics are unknown. The present study investigates clinical results obtained after perfusion method modification and temperature management during cardiopulmonary bypass (CPB).

**Methods:**

Between July 2010 and August 2012, 16 consecutive adult patients (mean age 50.0 yr ± 14.1 yr, range 25 yr to 73 yr, 12 males, 4 females) who presented with acute Stanford type-A aortic dissection underwent aortic arch replacement (total arch, n = 11; hemiarch, n = 5) under mild hypothermia (31.1°C ± 1.5°C) with right axillary and femoral artery perfusion.

**Results:**

The mean CPB time was 201 min ± 53 min, and the mean myocardial ischemic time was 140 min ± 42 min. The mean selective cerebral perfusion time was 80 min ± 16 min, and the mean lower-body circulatory arrest time was 20 min ± 13 min. No patient death occurred within 30 post-operative days. The following details were observed: new post-operative permanent neurologic deficit in 1 patient (6.3%), temporary neurologic deficit in 2 patients (12.5%), acute renal dysfunction (creatinine level > 230 umol/L) in 3 patients (18.8%) and mechanical ventilation > 72 h in 5 patients (31.2%).

**Conclusions:**

Aortic arch replacement for acute type-A aortic dissection under mild hypothermia with right axillary and femoral artery perfusion could be safely performed in the patient cohort.

## Background

Deep hypothermic circulatory arrest (DHCA) has gained wide application as a cornerstone for brain and other organ protection during aortic arch replacement. However, pure DHCA presents time limits, and some complicated cases may not be completed within the stipulated time. Therefore, application of various cerebral protection measures, including retrograde cerebral perfusion (RCP) and selective antegrade cerebral perfusion (SACP), is required. Randomised trials have revealed no evidence of significant metabolic or neurologic benefits of RCP [1-3]. SACP has recently been popularised [4-9] and offers a more physiological method of perfusion; the method extends current safety limits for aortic arch surgery. A unified understanding of the optimal temperature, however, is unavailable [10]. Whilst the safety of mild hypothermic circulatory arrest (HCA) with SACP has recently been reported [8], this technical modification has yet to gain widespread acceptance for aortic arch surgery. The present study aims to analyse the clinical effects of perfusion method modification and temperature management on aortic arch replacement; the safety of the method and its applicability in cerebral protection are also discussed.

## Methods

### Patients

Between July 2010 and August 2012, 16 consecutive adult patients (mean age 50.0 yr ± 14.1 yr, range 25 yr to 73 yr, 12 males, 4 females) who presented with acute Stanford type-A (DeBakey type I) aortic dissection underwent aortic arch replacement in the hospital selected in this study. Selective cerebral perfusion through the right axillary artery cannulation at mild hypothermia (31.1°C ± 1.5°C) was adopted. During selective cerebral perfusion, the lower body was simultaneously perfused through the femoral artery. Demographic and preoperative data of the patients are summarised in Table 1. One patient had a history of cerebrovascular accident, two patients required preoperative mechanical ventilation because of respiratory failure and one patient suffered from confusion because of temporary cerebral ischemia caused by dissection involving the innominate artery. This study was conducted with approval from the Ethics Committee of Sir Run Run Shaw Hospital. Written informed consent was obtained from all participants.

**Table 1:** Demographics for 16 patients undergoing aortic arch replacement

**Characteristic**	**n (%)**
Age (years ± SD)	50 ± 14.1
Male	12 (75.0%)
Female	4 (25.0%)
Body weight (Kg ± SD)	70.6 ± 10.6
Marfan syndrome	2 (12.5%)
Hypertension	8 (50.0%)
Smoking	6 (37.5%)
History of cerebrovascular accident	1 (6.3%)
Preoperative neurologic dysfuction	1 (6.3%)
Preoperative ventilation	2 (12.5%)

### Methods

**Figure 1:**
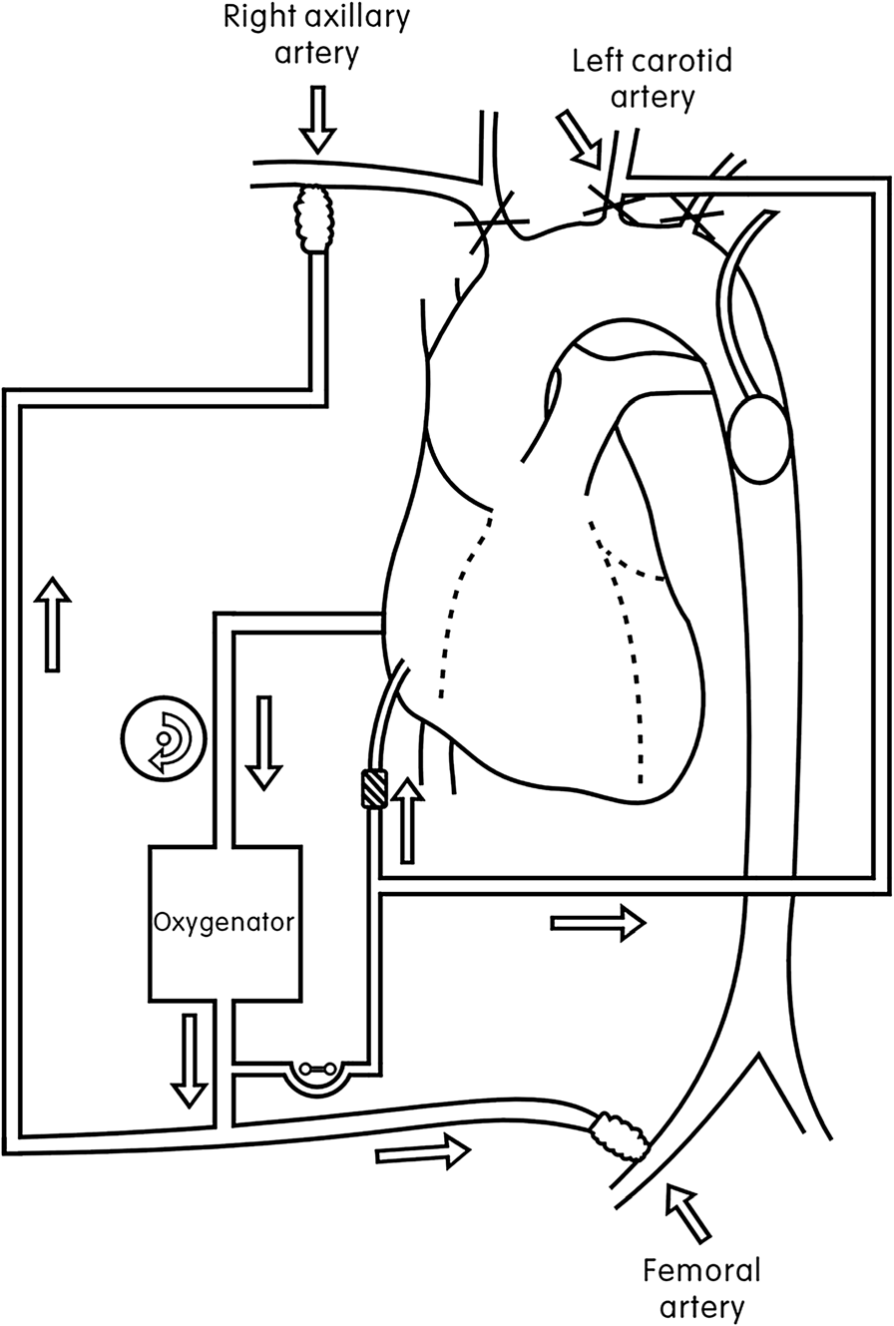
Cardiopulmonary bypass diagram.

Anaesthesia was induced and maintained in a standard manner. The patient was positioned in a supine position. The right radial artery and dorsalis pedis artery were cannulated for continuous blood pressure monitoring. A transesophageal echocardiographic probe was used for confirmation of the diagnosis and assessment of cardiac function; temperature probes for oesophageal and rectal temperature monitoring were also used. Two groups of doctors initially separated the right axillary and femoral arteries. After administration of a small dose of heparin n (heparin) (100 IU/kg), polyester vascular prostheses (Intervascular, La Ciotat, France) 8 and 10 mm in diameter were separately anastomosed to the right axillary and femoral arteries in an end-to-side fashion. Double arterial lines with a single centrifugal pump were adopted, and a Y joint was used to connect the axillary and femoral arteries separately. Perfusion of the upper and lower parts of the body was separately performed. The right atrium was cannulated with a two-stage venous cannula. Myocardial protection was achieved by antegrade and/or retrograde 4:1 cold-blood cardioplegia.Figure 1 illustrates the connections between the patient and the cardiopulmonary bypass (CPB) circuit. When the CPB circuit was started, the right axillary and femoral arteries were simultaneously perfused. During the slow-cooling phase, the ascending aorta was cut open, and the aortic root procedure was performed (Bentall operation or aortic valvuloplasty). After completing the aortic root procedure, three proximal brachiocephalic arteries were occluded when the nasopharyngeal temperature dropped to 30°C, and antegrade selective cerebral perfusion was initiated at a flow rate of 27.9 mL/kg/min ± 5.3 mL/kg/min (1971 mL/min ± 326 mL/min) through the right axillary artery. The right radial artery pressure was maintained at 60–80 mmHg, and the femoral artery perfusion was stopped at the same time. The aortic clamp was removed and the aortic arch was explored. A retrograde coronary sinus perfusion cannula (Medtronic, Watson, USA) was placed in the left common carotid artery to perfuse the left cerebral hemisphere, with a flow rate of 3.4 mL/kg/min ± 0.4 mL/kg/min (200–300 mL/min). For total arch replacement, the elephant-trunk technique and a four-branched graft (Intervascular) were employed. An intraoperative membranous stent graft (MicroPort, Shanghai, China) was first anchored to the inner wall of the descending thoracic aorta. Next, a 16 F urethral catheter was placed in the descending aorta. Water was injected through the injection port to occlude the descending aorta, after which femoral artery perfusion was resumed. A four-branched graft was separately anastomosed to the distal descending thoracic aorta, left common carotid artery and proximal ascending aorta, and the left subclavian artery was ligated. After de-airing, the aortic clamp was removed, and systemic perfusion was resumed. Finally, the last branch of the graft was anastomosed to the innominate artery. After completion of innominate artery anastomosis, right axillary artery perfusion was ceased. Femoral artery perfusion was continued until the CPB was stopped.

### Statistical analysis

All data were collected from medical charts, and continuous variables are expressed as mean ± standard deviation. Categorical variables are expressed as percentages using SPSS 13.0 software.

### Definitions

Temporary neurologic deficit (TND) was defined as the occurrence of at least one of the following symptoms despite normal computed tomography scans and resolution of all symptoms prior to discharge: post-operative confusion, agitation, delirium or transient parkinsonism. Permanent neurologic deficit (PND) was defined as the presence of either new stroke or coma. Post-operative renal dysfunction was defined as a creatinine level > 230 umol/L (twice the normal value).

## Results

Intraoperative data are provided in Table 2. The mean CPB time was 201 min ± 53 min, and the mean myocardial ischemic time was 140 min ± 42 min. The mean selective cerebral perfusion time was 80 min ± 16 min, and the mean lower-body circulatory arrest time was 20 min ± 13 min. Total arch replacement was performed in 11 patients (68.8%), whilst hemiarch replacement was performed in 5 patients (31.2%). Post-operative data are shown in Table 3. Mean chest tube drainage within the first 24 h after operation was 770 mL ± 626 mL. Red blood cell and plasma transfusions totalled 4.9 units ± 2.8 units and 1256 mL ± 430 mL, respectively, during operation and within the first 12 h after operation in the intensive care unit. Mean serum lactate before operation, after CPB and on post-operative day 1 were 1.7 mmol/L ± 1.1 mmol/L, 3.6 mmol/L ± 1.2 mmol/L and 2.2 mmol/L ± 1.5 mmol/L, respectively. No patient death occurred within 30 post-operative days. One patient (6.3%) suffered from PND (left hemiplegia caused by right cerebral infarction) but was able to look after himself in daily life after 6 months of rehabilitative training. Two patients (12.5%) suffered from temporary neurologic dysfunction, such as delirium. Neither paraplegia nor complication was related to the axillary or femoral artery cannulation. Post-operative renal dysfunction was noted in 3 patients (18.8%), 1 patient required temporary haemodiafiltration and another patient required permanent haemodialysis. CTA showed bilateral renal arteries were involved by dissection. Five patients (31.2%) required prolonged mechanical ventilation for over 72 h, amongst which 2 patients were preoperatively supported by mechanical ventilation, and 2 patients were reintubated. Amongst these 5 patients, 2 patients underwent tracheotomy on post-operative days 6 and 7 because of difficulties associated with their weaning from mechanical ventilation.

**Table 2:** Operative data and surgical procedures for 16 patients undergoing aortic arch replacement

**Operative data**	**Values**
Cardiopulmonary bypass time (min ± SD)	201 ± 53
Myocardial ischemic time (min ± SD)	140 ± 42
SCP time (min ± SD)	80 ± 16
Lower body circulatory arrest time (min ± SD)	20 ± 13
Nasopharyngeal temperature at SCP (°C ± SD)	31.1 ± 1.5
Rectal temperature at SCP (°C ± SD)	32.5 ± 1.4
SCP flowrate (ml/kg/min ± SD)	27.9 ± 5.3
Femoral artery perfusion flowrate (ml/kg/min ± SD)	33.8 ± 4.9
Hemiarch replacement (n/%)	5 (31.2%)
Total arch replacement (n/%)	11 (68.8%)
Aortic root procedure	
Bentall operation (n/%)	4 (25.0%)
Aortic valvuloplasty	7 (43.8%)

**Table 3:** Operative outcome of 16 patients undergoing aortic arch replacement

**Operative outcome**	**Values**
Chest tube drainage (ml/24 h ± SD)	770 ± 626
Intraop and postop 12 h RBC transfusion (unites ± SD)	4.9 ± 2.8
Intraop and postop 12 h plasma transfusion (ml ± SD)	1256 ± 430
Length of ICU stay (days ± SD)	7.5 ± 7.9
Ventilation > 72 hours (n/%)	5 (31.2%)
Tracheostomy (n/%)	2 (12.5%)
Renal dysfunction (n/%)	3 (18.8%)
Hemodiafiltration (n/%)	2 (12.5%)
Re-exploration for bleeding (n/%)	1 (6.3%)
Temporary neurologic deficit (n/%)	2 (12.5%)
Permanent neurologic deficit (n/%)	1 (6.3%)
30-days mortality (n/%)	0 (0%)

## Discussion

Aortic arch replacement for acute type-A aortic dissection remains a surgical challenge because of its relatively high mortality and morbidity. The mortality rate of the surgical treatment of acute Stanford type-A aortic dissection varies between 5% and 25%, and the rate of PND is between 5% and 10% [11]. Aortic arch replacements were performed in 16 consecutive acute Stanford type-A aortic dissection patients, and the technique of SACP with mild hypothermia (31.1°C ± 1.5°C) was applied. No deaths occurred in the patient cohort within 30 days after operation. In this study, a PND morbidity rate of 6.8% (1 patient) and a TND morbidity rate of 12.5% (2 patients) were obtained, which are considered satisfactory results.

SACP is probably the most widely used adjunctive cerebral protective technique to supplement HCA. However, the optimal temperature of SACP is undetermined. Animal research [11,12] has shown that cerebral oxygen consumption decreases by 50% if the temperature of the whole body is cooled to 28°C and that further cooling below 28°C does not effectively decrease cerebral oxygen consumption. In addition, regional cerebral blood flow obviously drops under cooling because of the opening of the arterio-venous shunt. Thus, moderate HCA (25°C to 28°C) with SACP has an inclination to popularity [13-15]. HCA with SACP prevents a series of adverse effects caused by deep hypothermia [15-17] and shows excellent clinical outcomes. Given continuous improvements in aortic surgical techniques and perfection of CPB equipment in recent years, several surgeons have completed aortic arch surgery under mild hypothermia (30°C to 32°C) with satisfactory results [6,11,12,18]. Andreas Zierer and colleagues [6] reported on 245 aortic arch operations completed using selective cerebral perfusion under mild hypothermia (30.5°C ± 1.4°C) in 2011 and obtained an operative mortality rate of 8% (*n* = 20) and post-operative PND and TND morbidity rates of 6% (n = 14) and 5% (*n* = 12), respectively.

Axillary artery cannulation for SACP has been widely used in recent years [19,20]. SACP was performed by anastomosing an 8 mm polyester vascular prosthesis to the right axillary artery. This method could optimise cerebral blood flow, facilitate precise pressure monitoring of the radial artery and decrease peripheral neuro-vascular injuries caused by direct cannulation. Pressure-controlled selective cerebral perfusion provides blood flow to the brain under nearly physiological ranges. A radial artery pressure of 60–80 mmHg was maintained by adjusting the flow rate of the centrifugal pump. According to previous reports [6,11], cerebral blood flow accounts for 20%–30% of the cardiac output under normal physiological status. Therefore, a mean flow of 27.9 mL/kg/min ± 5.3 mL/kg/min (1971 mL/min ± 326 mL/min) should sufficiently meet brain oxygen demands under mild hypothermia. Distal organ protection remains a difficult problem to address in patients requiring prolonged circulatory arrest in the lower body during selective cerebral perfusion [8]. The perfusion strategy employed in the present study is slightly different from that in most literature reports because it advocates routine femoral artery perfusion with occlusion of the descending thoracic aorta using an inflated urinary catheter, the mean flow is controlled to 33.8 mL/kg/min ± 4.9 mL/kg/min (2400 mL/min ± 396 mL/min) and the dorsalis pedis artery pressure is maintained at 40–80 mmHg. Some scholars believe that aortic dissection surgery carries the risk of false lumen perfusion during femoral artery cannulation. In the present study, direct cannulation was not performed; instead, a vascular prosthesis was anastomosed to the femoral artery under direct vision to ensure that the anastomosis is located in the true lumen and prevent false lumen perfusion. This method decreased the distal organ ischemic time. The circulatory arrest time was 20 min ± 13 min, shorter than that reported in most of the literature. Gilles and colleagues [21] reported that, using a similar method, circulatory arrest of the lower body could be nearly completely avoided during selective cerebral perfusion. These researchers completed aortic arch surgery for 29 patients under normothermic CPB and obtained a mortality rate of 6.8% (*n* = 2) and no neurologic complications. Our method of femoral artery perfusion for the lower body during selective cerebral perfusion is simple, feasible and has particular significance for complex surgery requiring prolonged circulatory arrest times (>60 min). Masashi Toyama and colleagues [12] reported 26 patients who underwent aortic arch surgery via mild HCA with SACP, amongst which 5 patients with prolonged circulatory arrest times (62 min ± 36 min) required haemodialysis post-operatively. In the present study, 2 patients required post-operative haemodialysis because of acute renal failure. One of these patients sustained renal hypoperfusion because of renal artery dissection. Femoral artery perfusion was temporarily stopped in two patients during selective cerebral perfusion because poor occlusion seriously interfered with the operating field. The circulatory arrest times of the two patients were 40 and 48 min.

Most scholars agree on unilateral perfusion for selective cerebral perfusion [13,22] and believe that unilateral perfusion can provide good cerebral protection as long as the integrity of the Circle of Willis is preserved. However, in theory, unilateral perfusion presents the risk of hypoperfusion in the left hemisphere, especially at mild hypothermia, and the safety of unilateral perfusion is still controversial. Wozniak and colleagues [23] once reported that out of 21 patients on whom unilateral perfusion at moderate hypothermia was performed, 2 suffered from temporary hemiplegia. Given that selective cerebral perfusion under mild hypothermia was performed in the present study and that judging the integrity of the Circle of Willis peri-operatively is difficult, bilateral perfusion was adopted to ensure safety. Bilateral perfusion involves inserting a retrograde coronary sinus perfusion cannula in the left common carotid artery at the beginning of selective cerebral perfusion and using the same roller pump for perfusion during the gap of cardioplegic solution. This method is easy to operate and manage, and the flow rate can be controlled to 200–300 mL/min (3.4 mL/kg/min ± 0.4 mL/kg/min).

The limitations of this study include the small number of patients surveyed and the lack of a control group. In addition, local condition restrictions prevented further monitoring of cerebral protection during the operation, such as by using transcranial Doppler to monitor middle cerebral artery flow velocity and cerebral emboli or jugular venous oxygen saturation measurement to monitor cerebral metabolism. We intend to address these issues in future work. Our proposed perfusion strategy also requires some time to anastomose the vascular prostheses to the right axillary and femoral arteries and may thus be unsuitable for acute aortic dissection patients with unstable preoperative haemodynamics or cardiac surgery centres that lack well-trained senior surgeons.

## Conclusion

SACP through the right axillary artery under mild hypothermia and simultaneous lower-body perfusion through the femoral artery is safe and feasible during aortic arch replacement. The technique provides satisfactory cerebral and distal organ protection and avoids deep hypothermia-related side effects. Further randomised prospective studies with a larger number of patients are necessary to confirm the present observations.

## Abbreviations

HCA: Hypothermic circulatory arrest; SACP: Selective antegrade cerebral perfusion; PND: Permanent neurologic deficit; TND: Temporary neurologic deficit; DHCA: Deep hypothermic circulatory arrest; RCP: Retrograde cerebral perfusion; CPB: Cardiopulmonary bypass; SPSS: Statistic package for social science; CTA: Computerized tomography angiography; SD: Standard deviation; SCP: Selective cerebral perfusion.

## Competing interests

The authors declare that they have no competing interests.

## Authors’ contributions

JG participated in clinical practice, contributed to conception, design, acquisition of data, drafting the manuscript, and revising it critically for important intellectual content. YW participated in clinical practice, contributed to design, analysis and interpretation of data, and helped in drafting the manuscript. JZ participated in clinical practice, contributed to design, helped in drafting the manuscript. JC participated in clinical practice. ZC participated in clinical practice. ZL helped in revising the manuscript critically for important intellectual content. XQ participated in clinical practice, contributed to design, and revising the manuscript critically for important intellectual content. All authors read and approved the final manuscript.
